# Awareness of cognitive decline trajectories in asymptomatic individuals at risk for AD

**DOI:** 10.1186/s13195-020-00700-8

**Published:** 2020-10-14

**Authors:** Federica Cacciamani, Luisa Sambati, Marion Houot, Marie-Odile Habert, Bruno Dubois, Stéphane Epelbaum, on behalf of the INSIGHT-PreAD study group, C. Audrain, A. Auffret, H. Bakardjian, F. Baldacci, B. Batrancourt, I. Benakki, H. Benali, H. Bertin, A. Bertrand, L. Boukadida, F. Cacciamani, V. Causse, E. Cavedo, S. Cherif Touil, P. A. Chiesa, O. Colliot, G. Dalla Barba, M. Depaulis, A. Dos Santos, B. Dubois, M. Dubois, S. Epelbaum, B. Fontaine, H. Francisque, G. Gagliardi, A. Genin, R. Genthon, P. Glasman, F. Gombert, M. O. Habert, H. Hampel, H. Hewa, M. Houot, N. Jungalee, A. Kas, M. Kilani, V. La Corte, F. Le Roy, S. Lehericy, C. Letondor, M. Levy, S. Lista, M. Lowrey, J. Ly, O. Makiese, I. Masetti, A. Mendes, C. Metzinger, A. Michon, F. Mochel, R. Nait Arab, F. Nyasse, C. Perrin, F. Poirier, C. Poisson, M. C. Potier, S. Ratovohery, M. Revillon, K. Rojkova, K. Santos-Andrade, R. Schindler, M. C. Servera, L. Seux, V. Simon, D. Skovronsky, M. Thiebaut, O. Uspenskaya, M. Vlaincu

**Affiliations:** 1 Institut du Cerveau, ICM, F-75013 Paris, France; 2 Inserm, U 1127, F-75013 Paris, France; 3 CNRS, UMR 7225, F-75013 Paris, France; 4 Sorbonne Université, F-75013 Paris, France; 5 Inria, APHP-Inria collaboration, Aramis-project team, Paris, France; 6 IRCCS, Istituto delle Scienze Neurologiche di Bologna, Bologna, Italy; 7 Dipartimento di Scienze Biomediche e NeuroMotorie (DIBINEM), Università di Bologna, Bologna, Italy; 8 Institute of Memory and Alzheimer’s Disease (IM2A), Centre of EXCELLENce of Neurodegenerative Disease (CoEN), National Reference Center for Rare or Early Dementias, Department of Neurology, Hôpital Pitié-Salpêtrière, AP-HP, 47-83 Boulevard de l’Hôpital, 75013 Paris, France; 9 CATI Multicenter Neuroimaging Platform (cati-neuroimaging.com), Paris, France; 10 Service de Médecine Nucléaire, Hôpital de la Pitié-Salpêtrière, AP-HP, Paris, France

**Keywords:** Preclinical Alzheimer’s disease, Awareness, Cognitive decline, Brain, Amyloid

## Abstract

**Background:**

Lack of awareness of cognitive decline (ACD) is common in late-stage Alzheimer’s disease (AD). Recent studies showed that ACD can also be reduced in the early stages.

**Methods:**

We described different trends of evolution of ACD over 3 years in a cohort of memory-complainers and their association to amyloid burden and brain metabolism. We studied the impact of ACD at baseline on cognitive scores’ evolution and the association between longitudinal changes in ACD and in cognitive score.

**Results:**

76.8% of subjects constantly had an accurate ACD (reference class). 18.95% showed a steadily heightened ACD and were comparable to those with accurate ACD in terms of demographic characteristics and AD biomarkers. 4.25% constantly showed low ACD, had significantly higher amyloid burden than the reference class, and were mostly men. We found no overall effect of baseline ACD on cognitive scores’ evolution and no association between longitudinal changes in ACD and in cognitive scores.

**Conclusions:**

ACD begins to decrease during the preclinical phase in a group of individuals, who are of great interest and need to be further characterized.

**Trial registration:**

The present study was conducted as part of the INSIGHT-PreAD study. The identification number of INSIGHT-PreAD study (ID-RCB) is 2012-A01731-42.

## Introduction

Patients with Alzheimer’s dementia often exhibit anosognosia; that is, they show little or no awareness of their progressive cognitive decline (e.g., [1]). According to longitudinal and cross-sectional studies, the awareness of cognitive decline (ACD) is not only impaired in patients with dementia, but also in about 50% of individuals with mild cognitive impairment (MCI) [2, 3]. Anosognosic MCI patients seem to have a greater risk of future progression to dementia than non-anosognosic ones (e.g., [4–6]).

Recently, a few studies have suggested the possibility of a very early reduction of the ACD in Alzheimer’s disease (AD), during its preclinical phase, without this being a clear anosognosia. In this initial phase of the disease, insidious neuropathological processes have started but have no or very slight impact on cognition [7–9]. In the transitional stage II of preclinical AD according to [9], individuals exhibit subtle cognitive decline, which corresponds to slight differences in cognitive efficiency and occasional memory lapses. Cognitive scores remain within age and educational norms but are probably close to the pathological testing threshold. An altered subjective perception of these initial cognitive changes could indicate an underlying early-stage AD pathology and allow for early diagnosis and patient management.

To date, very few studies have attempted to clarify the degree of ACD in individuals with preclinical AD. These studies have led to conflicting results, finding that both subjects with low ACD [10, 11] and those with marked cognitive complaints (or *hypernosognosia* [12]) had an increased risk of having positive AD biomarkers. Interestingly, a recent longitudinal study [13] found that these two conditions—low ACD and hypernosognosia—arrive consequently during the early phase of AD. Indeed, (i) a subset of individuals with preclinical AD who progressed to MCI showed hypernosognosia up to 1.6 years before MCI diagnosis; (ii) the participants with MCI who progressed to dementia showed low ACD, with clear anosognosia 3.2 years before the diagnosis of Alzheimer’s dementia.

In the present article, we aimed to investigate the potential usefulness of reduced ACD as an indicator of early-stage AD. This aim has been implemented through three objectives:

1. To track the temporality of the changes in the ACD during preclinical AD. More specifically, we aimed at identifying groups of participants who shared a similar longitudinal trajectory of the ACD and to characterize each group/trajectory according to their demographical data and AD neuroimaging markers at baseline (amyloid burden and brain metabolism);
2. To test the hypothesis that an early reduction in ACD is associated with progressive cognitive decline, by studying the impact of baseline ACD on changes in cognitive scores across the 3-year study period; and
3. To study the association between longitudinal changes in ACDI and longitudinal changes in cognitive scores, to clarify whether a certain pattern of ACD evolution is associated with a more marked cognitive decline.

## Methods and measures

### Participants

The INSIGHT-PreAD cohort has been described previously [14]. Briefly, 318 participants (and 318 study partners) were included in the study. They were French individuals between 70 and 85 years of age, with normal scores on Mini-Mental State Examination (MMSE ≥ 27), Clinical Dementia Rating scale (CDR = 0), and Free and Cued Selective Rating Test (FCSRT total recall score ≥ 41), who reported cognitive complaints at the study baseline (the subject answered *yes* to both of the following questions: Do you complain about your memory? Is this a regular complaint that has lasted for more than 6 months?). They had no evidence of monogenic AD mutations or other neurological disorders. The study partner had to be a person close to the subject, aware of potential recent changes in health and cognition. Each participant gave his/her informed consent, and Paris VI ethical committee approved the study protocol.

INSIGHT-PreAD is a prospective ongoing cohort study. When we performed the statistical analyses, the participants were undergoing their M54 or M60 visit. Only data up to M36 were analyzed in this article (7 timepoints), because the data of subsequent visits had not yet been fully collected and/or checked. However, at the time of the analyses,14 participants had been labeled as “decliners” (that is, exhibit at least two of the following changes over two consecutive evaluations 6 months apart: CDR increasing from 0 to 0.5 and/or an MMSE below 26 and/or a FCSRT total score below 40). The INSIGHT-PreAD protocol stipulated that participants would stop the follow-up as soon as they were classified as “decliners.” Three subjects were classified as “decliners” at the 24-month visit, three subjects at 36 months, one at 42 months, one at 54 months, and eight subjects at 60 months.

### Cognitive measures

We investigated the impact of baseline ACD on the evolution of the cognitive scores that are supposed to be most relevant to the study of ACD in early-stage AD.

All INSIGHT-PreAD participants performed cognitive screening tests every 6 months and underwent a comprehensive neuropsychological evaluation every 12 months.

On the one hand, impaired awareness is associated with a suboptimal online self-monitoring, error detection and disinhibition [15–17]. On the other hand, memory disorders prevent correct comparisons between current and past performance [18]. Therefore, we included (1) the Trail Making Test (TMT) B-A score, the Lexical and Semantic Fluency and the Frontal Assessment Battery (FAB), as measures of executive functioning; (2) the free recall and total recall scores from the FCSRT, an episodic memory test sensitive to hippocampal damage; and (3) the Mini-Mental State Examination (MMSE) as a measure of global cognitive functioning.

### Determination of the Awareness of Cognitive Decline Index (ACDI)

The procedure for identifying ACDI is reported in our previous publication [10].

In summary, the subjects and their study partners filled out two similar versions of the Healthy Aging Brain Care Monitor (HABC-M [19, 20]). This is a questionnaire asking how often, during the last 2 weeks, the participant has encountered certain difficulties in his/her everyday life. The questions are the same in the participant version and the study partner version and only asked slightly differently. For example, the first question in the participant version is “Over the past two weeks, how often *did you* have problems with judgment or decision making?”. In the informant version it is “Over the past two weeks, how often *did your loved one* have problems with judgment or decision making?”

Answers range from 0 (never) to 3 (very often). Since we aimed at studying awareness of *cognitive* decline, we only considered the HABC-M cognitive score, which is the sum of 6 items, and ranges from 0 to 18. The ACDI was determined by subtracting the HABC-M cognitive score obtained by the informant from that obtained by the subject. The ACDI ranged from − 18 to 18, where higher scores indicated heightened ACD (patient’s report > informant’s report), and lower scores, low ACD. The ACDI was computed at each visit (every 6 months) and treated as a continuous variable.

### Brain imaging

In the present study, we included baseline neuroimaging markers of AD.

All participants performed amyloid-PET imaging using ^18^F-AV-45 (^18^F-florbetapir; Amyvid™, Avid Radiopharmaceuticals) as a tracer. The standardized uptake value (^18^F-AV-45-SUV) was calculated in target regions (i.e., left and right precuneus, anterior cingulum, posterior cingulum, parietal, temporal, and orbitofrontal cortex) with the CATI pipeline (Centre d’Acquisition et de Traitement d’Images, https://cati-neuroimaging.com), and normalized to the cerebellum and pons, resulting in a SUV ratio (SUVr). In the present study, we considered the amyloid load as a continuous variable (mean ^18^F-AV-45 SUVr of the aforementioned regions) and as a dichotomic variable. To this end, the SUVr positivity threshold was 0.79, which was analogous to the threshold found using a method validated by Gael Chetelat in the IMAP study.

We also examined cortical glucose metabolism using fluorodeoxyglucose (FDG) PET. A metabolism index was calculated by averaging the FDG-SUVs of four bilateral regions of interest, whose metabolic changes are considered to be a “signature” of AD [21]: posterior cingulate cortex, inferior parietal lobule, precuneus, and inferior temporal gyrus. The pons was used as a reference region. The FDG-SUV has been included as a continuous variable.

More details about imaging data acquisition are available in previous works [14].

### Statistical analysis

For the first objective, we performed a Latent Class Linear Mixed Model (LCLMM [22]) to investigate heterogeneous trajectories of ACD, since they are expected in a cohort of memory-complainers. The LCLMM first identifies *G* classes of subjects who share a similar trend of evolution of the ACDI and then compares the classes. In order to find the adequate and clinically relevant number of classes *G*, we computed the model from one to three classes and selected the one which minimized the Bayesian Information Criterion (BIC). The mean of posterior probabilities and the percentage of posterior probabilities higher than 0.7 were computed. The evolution of the ACDI was modeled by the interaction between classes and visits. Using a multinomial logistic model, the baseline characteristics of each class (i.e., amyloid load, glucose metabolism, age, gender, and educational level) were compared to the class with the largest number of subjects. Normality of residuals and random effects as well as heteroskedasticity were checked visually. For this analysis, subjects with at least two timepoints of ACDI and with no missing baseline data (amyloid load, metabolism, age, gender, and education) were included.

We also performed generalized linear mixed effects models (GLMM) to evaluate the effect of the ACDI at baseline on changes in cognitive scores (objective 2) and the effect of longitudinal changes in ACD on the evolution of cognitive scores (objective 3). Link function was chosen for the underlying data generation mechanism with logit for binomial data and identity for continuous data. One GLMM was performed for each score, and then the Benjamini-Hochberg method was used to correct for multiple comparisons. For the second objective, we entered the following baseline variables as fixed effects: ACDI, age, gender, and educational level, visit, and the interaction between visit and ACDI, to test the impact of ACDI at baseline on changes in cognitive scores. All two-way interactions between these effects were tested independently and were added in the final model if significant. The participant was added as a random effect. For the third objective, we entered the following baseline variables as fixed effects: class, age, gender, and educational level and visit. All two-way interactions between these effects were tested independently and were added in the final model if significant. The participant was added as a random effect. Type II likelihood ratio tests were used to test each fixed effect and interaction. Cohen’s *f*^2^ were calculated, using the marginal *R*^2^ [23], for each effect to estimate their size. For this analysis, we only included subjects with at least two timepoints for each cognitive score, and with no missing data for ACDI at baseline or class, age at baseline, gender, and education. Baseline characteristics were compared between subjects included and excluded in the analysis using the *χ*^2^ test for categorical variables and Student’s *t* test for continuous variables.

A *p* value < 0.05 was considered significant. Statistical analyses were performed using R3.6.1. The packages lme4 (1.1-21) and LCMM (1.8.1) were used to perform LMM and LCLMM, respectively.

## Results

### First objective: study of trends of ACDI evolution and their association to AD neuroimaging markers

**Fig. 1:**
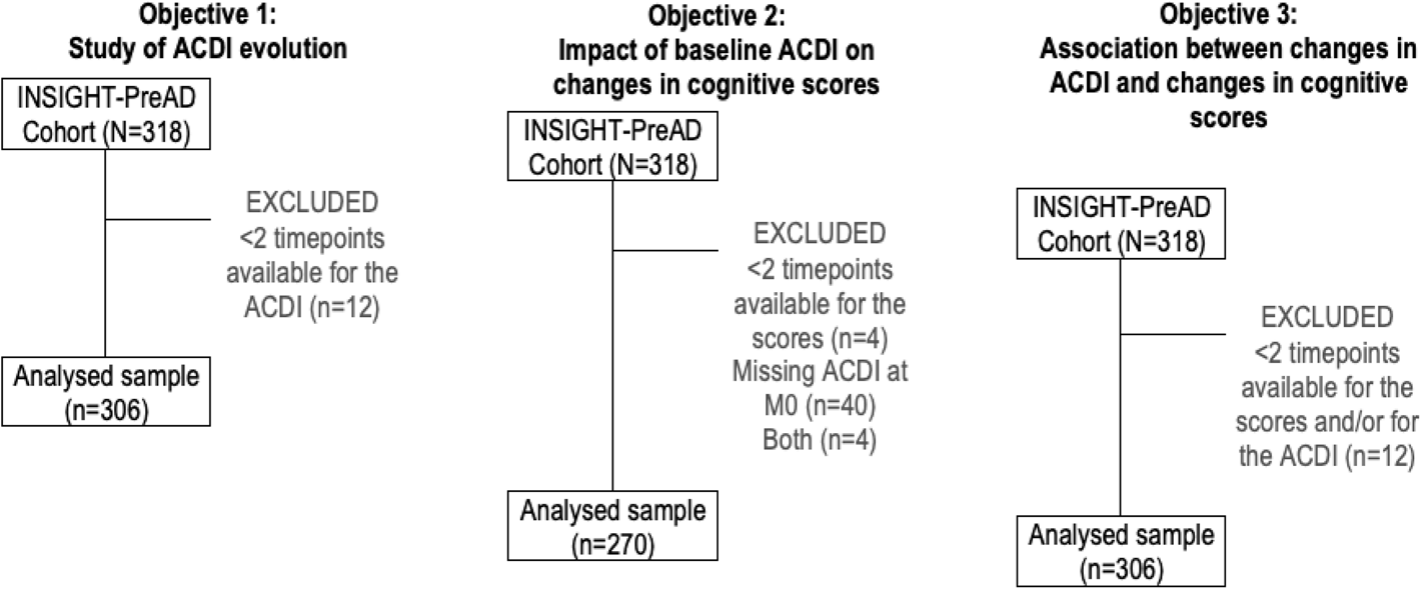
Sample selection for the three objectives

The longitudinal evolution of ACDI was studied in 306 out of 318 subjects. Indeed, we excluded 12 subjects who had only one ACDI available (*n* = 6) or none (*n* = 6) (Fig. 1).

The baseline characteristics of the included subjects are presented in Table 1. Mean age was 76.0 (SD = 3.4); subjects were mostly women (62.8%) and had high educational level (69% of the sample had at least a high school diploma).

**Table 1:** Baseline characteristics of the participants included in the analysis for the objective 1

	All subjects^¥^ *N* = 306 (96.23%)
Age [years; M ± SD]	75.95 ± 3.43
Gender [female; *n* (%)]	192 (62.75%)
Education [high^§^; *n* (%)]	211 (68.95%)
ACDI [M ± SD]	1.44 ± 2.92
HABC-M cognitive score (subject) [M ± SD]	3.32 ± 2.79
HABC-M cognitive score (informant) [M ± SD]	1.84 ± 2.21
MMSE [M ± SD]	28.67 ± 0.95
CDR [= 0; *n* (%)]	306 (100%)
FSCRT free recall [M ± SD]	30.15 ± 5.35
FSCRT total recall [M ± SD]	46.10 ± 1.97
FAB [M ± SD]	16.44 ± 1.66
TMT B-A [s; M ± SD]	47.81 ± 35.40
Lexical fluency [M ± SD]	22.46 ± 5.91
Semantic fluency [M ± SD]	31.44 ± 7.16
APOE [presence of ε4; *n* (%)]	60 (19.61%)
Amyloid load [^18^F-AV-45 SUVr; M ± SD]	0.78 ± 0.19
Brain glucose metabolism [^18^F-FDG SUV; M ± SD]	2.45 ± 0.25

*ACDI* Awareness of Cognitive Decline Index, *HABC-M* Healthy Aging Brain Care – Monitor, *MMSE* Mini-Mental State Examination, *CDR* Clinical Dementia Rating, *FCSRT* Free and Cued Selective Reminding Test, *FAB* Frontal Assessment Battery, *TMT* Trail Making Test, ^*18*^*F-AV-45 SUVr* florbetapir standardized uptake value ratio (index of amyloid deposition), ^*18*^*F-FDG SUV* fluorodeoxyglucose standardized uptake value (metabolic index)

^¥^Subjects included in the analysis were those with age, gender, educational level, ^18^F-AV-45 SUVr and ^18^F-FDG SUV available at baseline, and at least two timepoints for the ACDI

^§^Equal to or higher than high-school diploma (≥ 12 years)

**Fig. 2:**
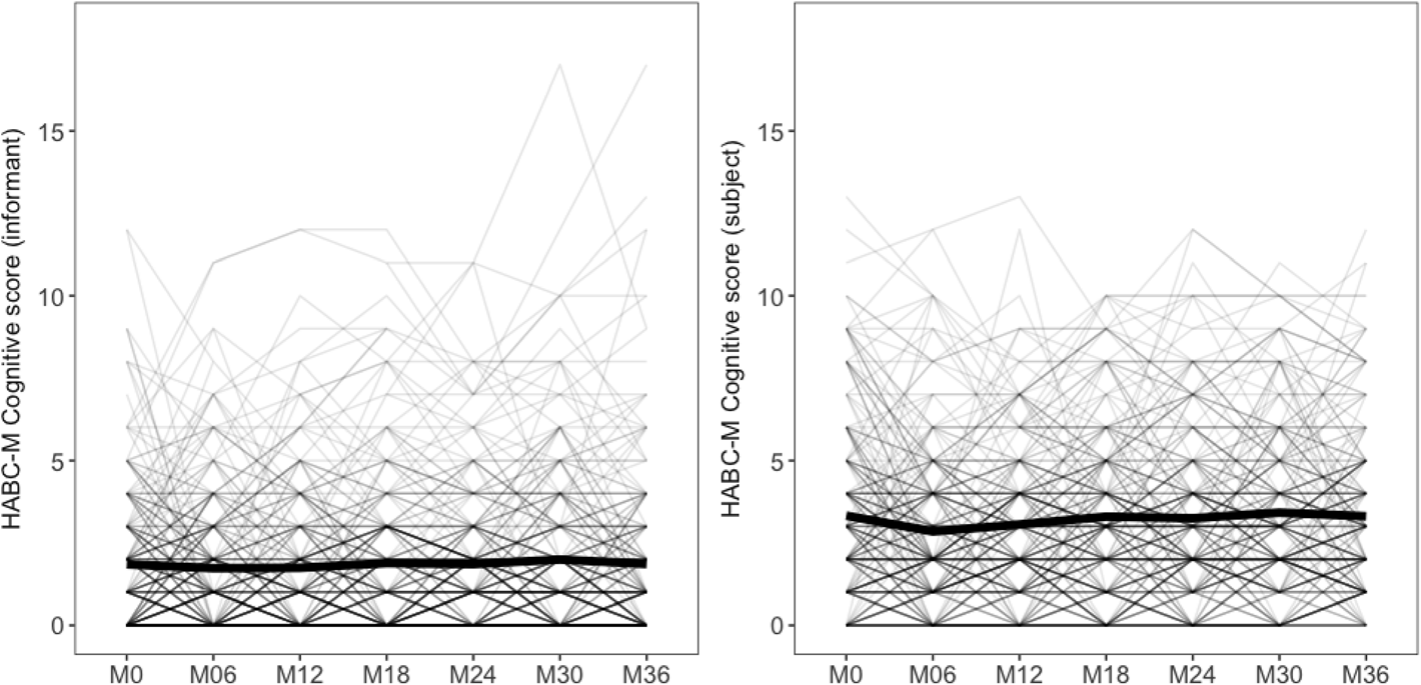
Trajectories of the subjects’ and informants’ HABC-M scores in the whole sample

The longitudinal changes of the subjects’ and informants’ HABC-M scores are presented in Fig. 2.

In each class, at least 70% of the subjects had at least 6 timepoints.

The three-class LCLMM provided the best fit with a BIC = 8287.2, compared to 8384.0 for the two-class LCLMM and 8626.7 for the one-class LCLMM. The three classes were distinct, with class 1 mean posterior probabilities of 0.91 belonging to class 1, class 2 mean posterior probabilities of 0.96 belonging to class 2, and class 3 mean posterior probabilities of 0.94 belonging to class 3. Moreover, more than 90% of the subjects in each class had a posterior probability higher than 0.7.

**Fig. 3:**
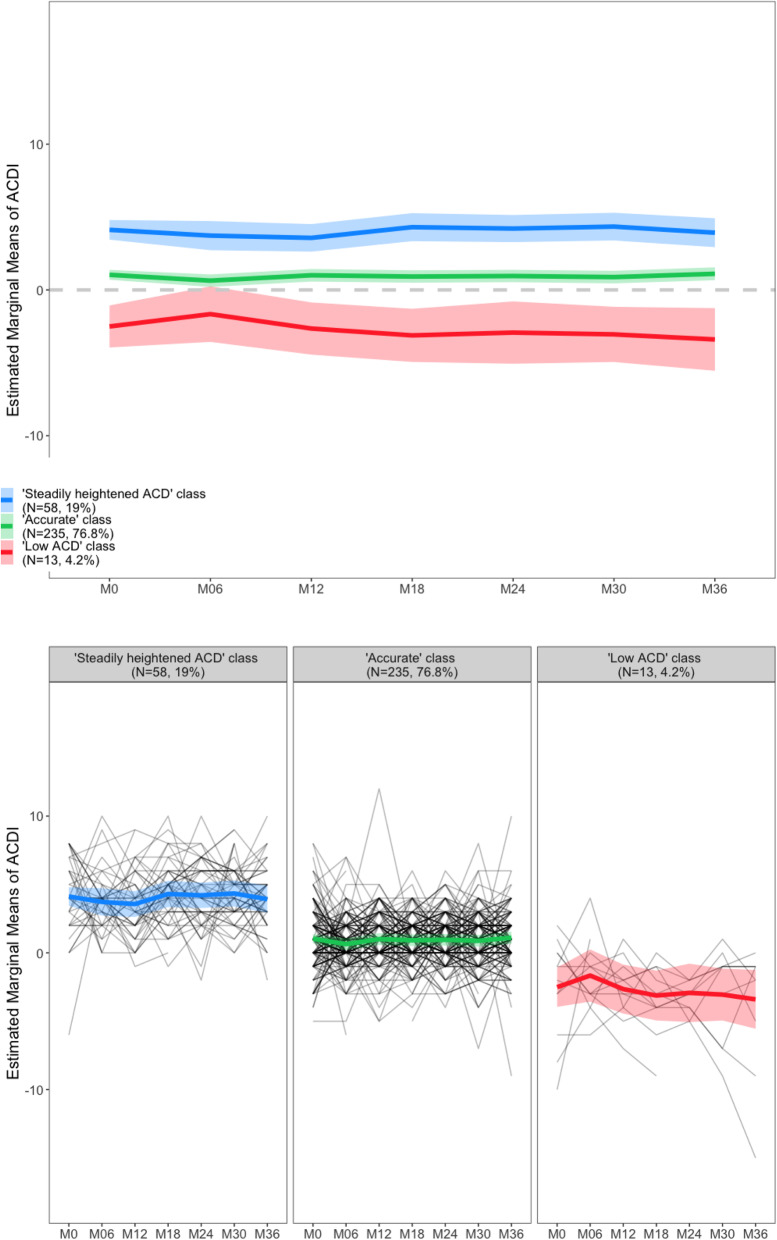
Evolution of the ACDI across the 36 months of study in the three classes of subjects identified by the LCLMM (objective 1)

The three trajectories identified by the LCLMM are presented in Fig. 3. Class 1 included 58 subjects (18.95% of the sample) having a relatively higher positive ACDI, meaning that these participants persistently reported more cognitive difficulties than their study partner did. We refer to these participants as belonging to the “steadily heightened ACD” class.

Class 2 included 235 subjects (76.8% of the sample) with an ACDI of around 0, indicating a good match between the subject’s and the informant’s assessments (“accurate ACD” class). The ACDI of this class remained unchanged over time.

Class 3 included 13 subjects (4.25% of the sample) with a relatively low ACDI (below zero), which means that these participants expressed less difficulties compared to their informant. In this class, ACDI slightly increased at 6 months and then tended to decline. We refer to this group as the “low ACD” class.

We compared the characteristics of classes 1 and 3 with those of class 2, which was chosen as the reference (Table 2). Indeed, this was the numerically largest class, and the average ACDI was constantly around 0 in this class, indicating that the subject and his/her study partner had similarly assessed the subject’s cognitive functioning.

**Table 2:** Comparison between class 2 (reference) and both classes 1 and 3

	Class 1 (“heightened ACD”, *n* = 58) vs class 2 (reference, *n* = 235)		Class 3 (“low ACD”, *n* = 13) vs class 2 (reference, *n* = 235)	
	OR ± se	*p* value	OR ± se	*p* value
Intercept	0.03 ± 0.12	0.3566	1867.11 ± 14,807.31	0.3422
Age at baseline	1.02 ± 0.04	0.6175	0.87 ± 0.08	0.1325
Gender [female]	1.47 ± 0.46	0.2123	4.66 ± 3.32	0.0307*
Education [high^§^]	1.73 ± 0.53	0.0783	0.28 ± 0.30	0.2389
Amyloid load [^18^F-AV-45 SUVr]	0.99 ± 0.85	0.9950	57.18 ± 69.93	0.0009*
Amyloid status [A*β*- subjects]	0.84 ± 0.30	0.6262	3.70 ± 2.31	0.0357*
Brain metabolism [^18^F-FDG SUV]	1.03 ± 0.62	0.9560	0.18 ± 0.26	0.2289

Class 2, with stable evolution and matching subject’s and informant’s ratings of decline, was the reference class. For categorical variables, category in brackets was the reference

^*18*^*F-AV-45 SUVr* florbetapir standardized uptake value ratio (index of amyloid deposition). Effect adjusted for age, gender, education, and FDG load

^*18*^*F-FDG SUV* fluorodeoxyglucose standardized uptake value (metabolic index)

^§^Equal to or higher than high-school diploma (≥ 12 years)

*OR* odd ratio, *se* standard errors

* Statistically significant difference (*p* < 0.05)

Compared to class 2, individuals in class 3 (low ACDI) had higher amyloid burden (OR ± SE 57.2 ± 69.9; *p* = 0.0009), were mostly amyloid-positive (OR ± SE 3.70 ± 2.31; *p* = 0.0357), and were mostly men (OR ± SE 4.7 ± 3.3; *p* = 0.0307). No statistical difference was found in terms of age, educational level, and brain metabolism between these two classes. Class 3 includes 3 subjects (23.1% of this class) whose cognition became abnormal after 24, 42, and 54 months of follow-up, respectively.

No statistically significant difference was found when comparing classes 1 and 2 in terms of age, gender, educational level, amyloid burden, and brain metabolism. Class 1 included 1 subject labeled as a “decliner” at the 36-month visit, representing 1.72% of this class. Class 2 included 10 decliners (*n* = 2 after 24 months of follow-up; *n* = 8 after 60 months), which represents 4.25% of this class.

### Second objective: impact of ACDI at baseline on changes in cognitive scores

The second objective of this study was to investigate if the ACDI at baseline had an impact on changes in cognitive scores of interest across the 3-year study period. To do so, we only analyzed the participants who had ACDI available at baseline and at least two timepoints for one or more cognitive score(s) (*n* = 270) (Fig. 1). No significant difference was found when comparing subjects included and excluded from this analysis, in terms of demographic variables, cognitive scores, and biomarkers (all *p* > 0.156). The mean age was 75.9 (SD = 3.4) and subjects included were mostly women (61.9%) and had high educational level (68.5%).

Concerning the GLMM, the final models also included interactions between gender and visit, educational level and age, and ACDI and age, in addition to the effects stated in the statistical analysis paragraph. Marginal *R*^2^s, based on the GLMM fixed part, were very low from 0.05 (MMSE model) to 0.12 (Lexical fluency model). There was a significant overall change to all cognitive scores, except for the TMT B-A (*p* = 0.1449). However, this effect was very small (Cohen’s *ƒ*^2^ ranging from 0.004 to 0.034 for the different scores).

The main effect of the ACDI at baseline on the evolution of the scores was not significant for any test (all *p* > 0.6236).

The interaction between age and ACDI had a significant effect on the average evolution of Semantic Fluency score (*p*_non-corrected_ = 0.0443) and TMT B-A score (*p*_non-corrected_ = 0.0166), but the effect size was small (Cohen’s *ƒ*^2^ = 0.009 for Semantic Fluency score; Cohen’s *ƒ*^2^ = 0.015 for TMT B-A score), and the two effects did not survive the correction for multiple comparisons (*p* = 0.1160 for TMT B-A score; *p* = 0.1550 for Semantic Fluency score).

The effect of the interaction between timepoint and ACDI on the scores was not significant (all *p* > 0.1649).

### Third objective: association between trends of changes in the ACD and changes in cognitive scores

We found no significant effect of the variable “class” (i.e., the trend of evolution of the ACDI: “steadily heightened ACD,” “accurate,” “low ACD”) on the MMSE, FCSRT free and total recall, TMT B-A, and Semantic and Lexical fluency (all *p* > 0.0554; all *p*_corrected_ > 0.1523; all Cohen’s *ƒ*^2^ < 0.015). Only the “low ACD” class had a significantly higher FAB score than the “accurate” class (*p* = 0.0260), but this effect did not survive the correction for multiple comparisons using the Benjamini-Hochberg method (*p*_corrected_ = 0.0911), and the effect size was small (Cohen’s *ƒ*^2^ = 0.004).

## Discussion

It has recently been proposed that low ACD (patient’s complaint < informant’s complaint) may serve as a marker of early-stage AD. This is a recent field of research and the studies available are few, especially the longitudinal ones. Thus, this is one of the first studies to appreciate the longitudinal evolution of the ACD in a population of asymptomatic individuals at risk for AD, due to their age, cognitive complaints, and amyloid burden (where appropriate).

In our sample, we identified three patterns of evolution of the ACD across the 3 years of study.

Most subjects (around 77%) consistently expressed an accurate assessment of their own cognitive functioning, that is, the cognitive difficulties reported by the subject were comparable to those reported by his/her study partner (ACDI consistently around 0). This represents an accurate ACD.

The other two classes represented two forms of altered ACD (in agreement with the model proposed by Dalla Barba et al. [24]).

On the one hand, around 19% of participants consistently belonged to the “steadily heightened ACD” class, since they persistently reported more cognitive difficulties than their study partner did. These individuals were comparable to those with accurate ACD in terms of demographic characteristics and AD neuroimaging markers, which means that subjects with persistent cognitive complaints do not have a greater risk of having positive AD markers. Moreover, only one of these participants (1.72%) was tagged as decliner during the follow-up. This is consistent with previous studies, such as [10, 11]. Indeed, these individuals may subjectively experience a cognitive decline that is actually normal for their age [25], or due to conditions other than AD, such as sleep disturbances [26], or medications that impact cognition [27]. Anxiety and fear of potentially having dementia may play a central role in determining the emergence of cognitive complaints [28]. People close to the patient generally do not notice the decline described in these situations, resulting in the patient’s heightened ACD. The condition of *heightened ACD* cannot therefore be considered as specific to AD.

On the other hand, approximately 4% of subjects consistently had a low ACD, as they reported fewer cognitive difficulties than estimated by their study partner. This means that the ACD could already be reduced in asymptomatic elderly individuals at risk for AD and is particularly interesting if we consider that we studied a population of memory complainers. Thus, it seems that these two phenomena (low ACD and cognitive complaints) can coexist in the same individual, despite being apparently opposed. Indeed, individuals from the “low ACD” group were all complaining of a certain degree of cognitive difficulties, still underestimating them when compared to an informant.

These subjects showed higher amyloid burden than those with normal ACD and, as a consequence, a higher risk of developing AD [29], consistent with what has been found by [10, 11]. Around 1/4 of them (*n* = 3) were tagged as decliners during follow-up, a fraction which is qualitatively larger than in the other classes (although no statistics were performed due to the low number of decliners).

Interestingly, we found no difference between individuals with low and normal ACD in terms of brain metabolism. This suggests that the reduction of ACD would be associated with amyloid accumulation prior to neurodegeneration, of which brain hypometabolism is a marker [30]. This would be consistent with previous evidence concerning how the temporal sequence of imaging markers reflecting the pathological cascade of AD [31, 32] and underlines how this symptom could occur very early in the course of the disease.

It should be noted, however, that we examined the mean glucose metabolism in AD “signature” regions [21] and that this could mask regional differences. Indeed, we believe that ACD reduction could be associated with reduced local brain metabolism (in particular, in the frontal lobe) and not in other brain areas. Additional studies should be conducted to explore these regional differences, both in the glucose metabolism and in amyloid accumulation.

Another key result of this study is that individuals with low ACD were mostly men, consistent with previous evidence. For instance, a study conducted in the context of brain injuries found that men were less aware of their brain injury-related deficits compared to women [33]. A socio-cultural process could be responsible for the observed gender-related differences. It is well-known that men and women learn different gendered attitudes and behaviors from cultural values and norms, which result in different expectations regarding their social role. These experiences also include health-related behaviors. Research findings have been strikingly consistent in showing that men, as a group, are more likely to avoid seeking help for physical and mental health problems. Indeed, seeking help is associated with behaviors such as admitting vulnerability and showing weakness, leading men to experience a gender-role conflict (see [34] for a review). For the same reasons, when men do seek medical help, their behaviors may be different compared to women. For instance, they may ask fewer questions than women do [35]. We believe that these socio-cultural factors could explain why the group of subjects who underestimated their cognitive difficulties were mostly men. However, further studies should investigate an alternative explanation, namely that low ACD in the preclinical phase could be a better indicator for future progression to AD in men than women, due, for instance, to biological differences.

The longitudinal trajectory of the ACDI showed an increase at 6 months and then a tendency to decline, in those with consistently low ACD. We believe that the ACD is likely to be amplified in the presence of the earliest subtle cognitive difficulties (i.e., SCD), and later, in the preclinical phase, it would begin to decrease and becomes a clear anosognosia in AD dementia.

In the present study, we also explored (objective 2) whether an early reduction in ACD could anticipate a progressive cognitive impairment. We found that the changes in cognitive scores over 3 years did not depend on baseline ACD, meaning that the subjects who had lower ACD showed no more marked cognitive decline than the other subjects. We also found (objective 3) that the three patterns of evolution of the ACDI identified were not associated with different trends of changes in cognitive function. Indeed, this is a cohort of cognitively intact individuals, with a fraction of them likely being in an early (preclinical) stage of AD. On average, their cognitive scores remained stable during follow-up. Therefore, a lack of association between baseline or longitudinal ACD and the evolution of cognitive scores was somewhat expected, consistently with what we found in [10] in the same cohort. We believe that if the follow-up had been longer, we would have identified an association between ACD and cognitive scores’ evolution (i.e., those with an early low ACD would experience a more marked cognitive decline).

Taken together, our findings suggest that the caregiver/study partner report should be systematically collected, both in clinical settings for diagnostic purposes and in research settings to better select the subjects for inclusion in studies targeting early-stage AD.

However, the informant’s report should be collected in order to compare it with the participant’s (or patient’s) report. We believe that the informant’s report alone may not be as informative to the researcher or physician as the subject-informant discrepancy. Indeed, the fact that the subject reports more cognitive difficulties than the informant makes it less likely that he/she is affected by AD; on the contrary, the fact that he/she underestimates his/her cognitive difficulties should indicate a suspected neurodegenerative disease, probably of the Alzheimer’s type.

### Limitations

While this study was conducted on a single-center cohort, with highly standardized clinical assessment, neuropsychological testing, and imaging acquisition procedures, it should be noticed that our results may possibly be biased by the high average education level of participants and the over-representation of women, both of which could limit the generalization of our findings.

## Conclusion

To conclude, ACD may start to decrease in the very early stages of AD, especially in a certain group of individuals who need to be further characterized through additional studies. This group is of great interest because it is more at risk of being affected by AD than other individuals. Indeed, a progressive decline of ACD, but not the persistent presence of cognitive complaints, was shown to be associated with greater amyloid deposition. The presence of an informant is therefore strongly recommended both in clinical practice and in research trials. Indeed, this can be useful to orient the clinician towards making a timely diagnosis of AD. Inclusion criteria of studies investigating preclinical AD should also take into consideration this evidence.

## Data Availability

Data may be made available upon reasonable request from qualified investigators.

## Abbreviations

ACD: Awareness of cognitive decline
MCI: Mild cognitive impairment
MMSE: Mini-Mental State Examination
CDR: Clinical Dementia Rating
FCSRT: Free and Cued Selective Reminding Test
TMT: Trail Making Test
FAB: Frontal Assessment Battery
ACDI: Awareness of Cognitive Decline Index
HABC-M: Healthy Aging Brain Care Monitor
^18^F-AV-45: ^18^F-florbetapir; Amyvid™, Avid Radiopharmaceuticals
^18^F-AV-45-SUV: ^18^F-florbetapir standardized uptake value
CATI: Centre d’Acquisition et de Traitement d’Images
FDG-PET: Fluorodeoxyglucose positron emission tomography
LCLMM: Latent Class Linear Mixed Model
BIC: Bayesian Information Criterion
GLMM: Generalized linear mixed effects models
